# Hepatitis C Virus Envelope Glycoproteins: A Balancing Act of Order and Disorder

**DOI:** 10.3389/fimmu.2018.01917

**Published:** 2018-08-24

**Authors:** Samantha A. Yost, Yuanyuan Wang, Joseph Marcotrigiano

**Affiliations:** ^1^Department of Chemistry and Chemical Biology, Center for Advanced Biotechnology and Medicine, Rutgers University, Piscataway, NJ, United States; ^2^Laboratory of Infectious Diseases, National Institute of Allergy and Infectious Diseases, National Institutes of Health, Bethesda, MD, United States

**Keywords:** hepatitis C virus, envelope glycoprotein, vaccine design, HCV, E2, E1

## Abstract

Chronic hepatitis C virus infection often leads to liver cirrhosis and primary liver cancer. In 2015, an estimated 71 million people were living with chronic HCV. Although infection rates have decreased in many parts of the world over the last several decades, incidence of HCV infection doubled between 2010 and 2014 in the United States mainly due to increases in intravenous drug use. The approval of direct acting antiviral treatments is a necessary component in the elimination of HCV, but inherent barriers to treatment (e.g., cost, lack of access to healthcare, adherence to treatment, resistance, etc.) prevent dramatic improvements in infection rates. An effective HCV vaccine would significantly slow the spread of the disease. Difficulties in the development of an HCV culture model system and expression of properly folded- and natively modified-HCV envelope glycoproteins E1 and E2 have hindered vaccine development efforts. The recent structural and biophysical studies of these proteins have demonstrated that the binding sites for the cellular receptor CD-81 and neutralizing antibodies are highly flexible in nature, which complicate vaccine design. Furthermore, the interactions between E1 and E2 throughout HCV infection is poorly understood, and structural flexibility may play a role in shielding antigenic epitopes during infection. Here we discuss the structural complexities of HCV E1 and E2.

## Introduction

Hepatitis C virus (HCV) presents with mild symptoms; as an acute illness that resolves within weeks; or a lifelong, chronic infection that can lead to cirrhosis, liver cancer, and, if left untreated, death. End-stage, liver disease caused by chronic HCV infection is the leading cause of liver transplantation in the United States, Europe, and Japan (1–3). According to the World Health Organization, there were 1.75 million new HCV infections and 71 million people living with chronic HCV infection worldwide in 2015. Intravenous drug use and unsafe healthcare practices are responsible for a majority of new infections, contributing heavily to the doubling of HCV incidence in the United States between 2010 and 2014 (4, 5).

Despite FDA approval of several direct acting antiviral (DAA) treatments for HCV with very high success rates (>90%) for all genotypes, many at-risk groups are still spreading infection faster than they are being cured (5–8). Chronic HCV prevalence is about 1% of the total world population, but is much higher in many areas where healthcare is not widely accessible. Mongolia, Uzbekistan, Egypt, and Gabon, for example, have HCV prevalence ranging from 4 to 7% (5) and specific populations in the Nile Delta and Upper Egypt can have infection rates as high as 28%, varying heavily based on socioeconomic status (9). The poorest and least educated in Egypt have the highest HCV infection rates and simply do not have the means to receive treatment. Furthermore, intravenous drug use accounts for about 23% of new HCV infections (10). Populations of intravenous drug users worldwide must overcome several barriers to treatment such as high cost, access to healthcare, compliance, and fear of being discovered as a drug user (8). After a successful course of treatment however, if the patient continues engaging in risky behaviors, they are still at risk to be re-infected. These factors prevent a dramatic improvement in HCV infection rates worldwide. Therefore, it seems unlikely that DAAs alone will eliminate HCV infection without an effective vaccine.

HCV is an enveloped virus containing a positive-sense, single stranded RNA genome. The lipid envelope, derived from the host membrane, is embedded with two type I transmembrane proteins, envelope glycoproteins E1 and E2, which form a heterodimer (11). HCV particles are uniquely associated with lipids and apolipoproteins, which play a role in proper formation and function of secreted virions (12–19). These associations give viral particles an overall low buoyant density (16). The E1/E2 heterodimer is responsible for viral entry from recognition of host cell receptors to membrane fusion. Initial host-virus attachment interactions are through glycosaminoglycans and low-density lipoprotein receptor (20). Several receptors have a necessary role in entry such as claudin-1, occludin, CD81, and scavenger receptor class B type 1, mainly through interaction with E2, although the role of E1 is not fully understood (21–24). E1 and E2 are on the surface of the virion, available for host immune recognition, and are ideal for studies in immunogenicity ultimately leading to vaccine design; however, the conformation of the E1/E2 heterodimer and its interactions have not been well characterized throughout the various stages of virus assembly, host cell attachment, and membrane fusion. High quality, fully glycosylated and disulfide-linked envelope glycoproteins have proven to be difficult to produce in large quantities for biophysical study until recently.

## Envelope glycoprotein E1

**Figure 1:**
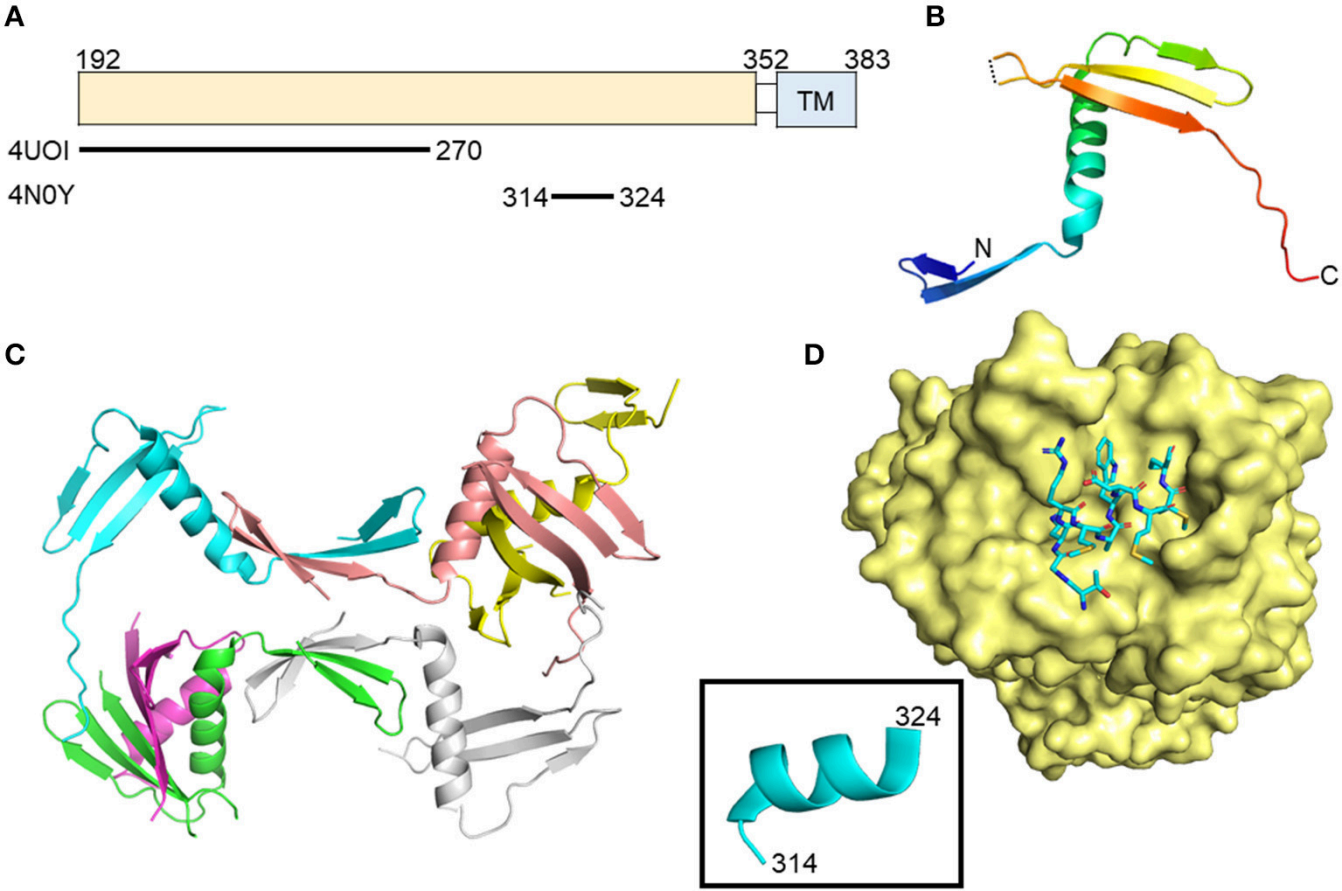
Crystal structures of the N-terminal domain of E1 and an E1 peptide in complex with antibody. **(A)** Linear diagram of E1 glycoprotein. The crystallized E1 constructs and PDB IDs are shown below the diagram. **(B)** N-terminal domain structure of E1 monomer (PDB ID: 4UOI). **(C)** The six molecules of the asymmetric unit of the E1 N-terminal domain. **(D)** Structure of E1 peptide (aa314–324) in complex with antibody IGH526 (PDB ID: 4N0Y). The surface of antibody IGH526 is colored yellow, with the E1 peptide colored according to atom type (light blue, red, orange, and dark blue for carbon, oxygen, sulfur and nitrogen, respectively). The E1 peptide is further shown as ribbon structure in the box.

The exact role(s) of E1 during entry, egress, and immune escape is not fully understood (21–24). It has an N-terminal ectodomain of approximately 160 amino acids and exists as a trimer on the surface of cell culture-produced HCV particles, driven by interactions in the E1 C-terminal transmembrane region (25) (Figure 1). E1 may aid in recognition of hepatocytes through interactions with apolipoproteins, particularly ApoE, which further interacts with cell surface heparin sulfate during early attachment (26, 27). Structural data of N-terminal 79 amino acids of HCV E1 (nE1) was determined by X-ray crystallography (28). This structure showed a covalently linked, domain-swapped homodimer with nE1 forming 16 amino-acid α-helix flanked by a β-hairpin N-terminally and a three-stranded antiparallel β-sheet C-terminally (Figures 1A–C). The N-terminus of E1 does not resemble a class II fusion protein as hypothesized, or any other fusion protein conformation, despite having a fusion peptide-like domain (29); however, the published structure may be in a post-fusion conformation as crystals were obtained at a low pH. The cross-neutralizing, anti-E1 antibody IGH526 was shown to bind to an α-helical epitope (residues 314–324) predicted to be highly flexible in molecular dynamics simulations (Figure 1D) (30). This is the first E1 antigenic epitope structure described, and may assist in future vaccine design.

## Envelope glycoprotein E2

**Figure 2:**
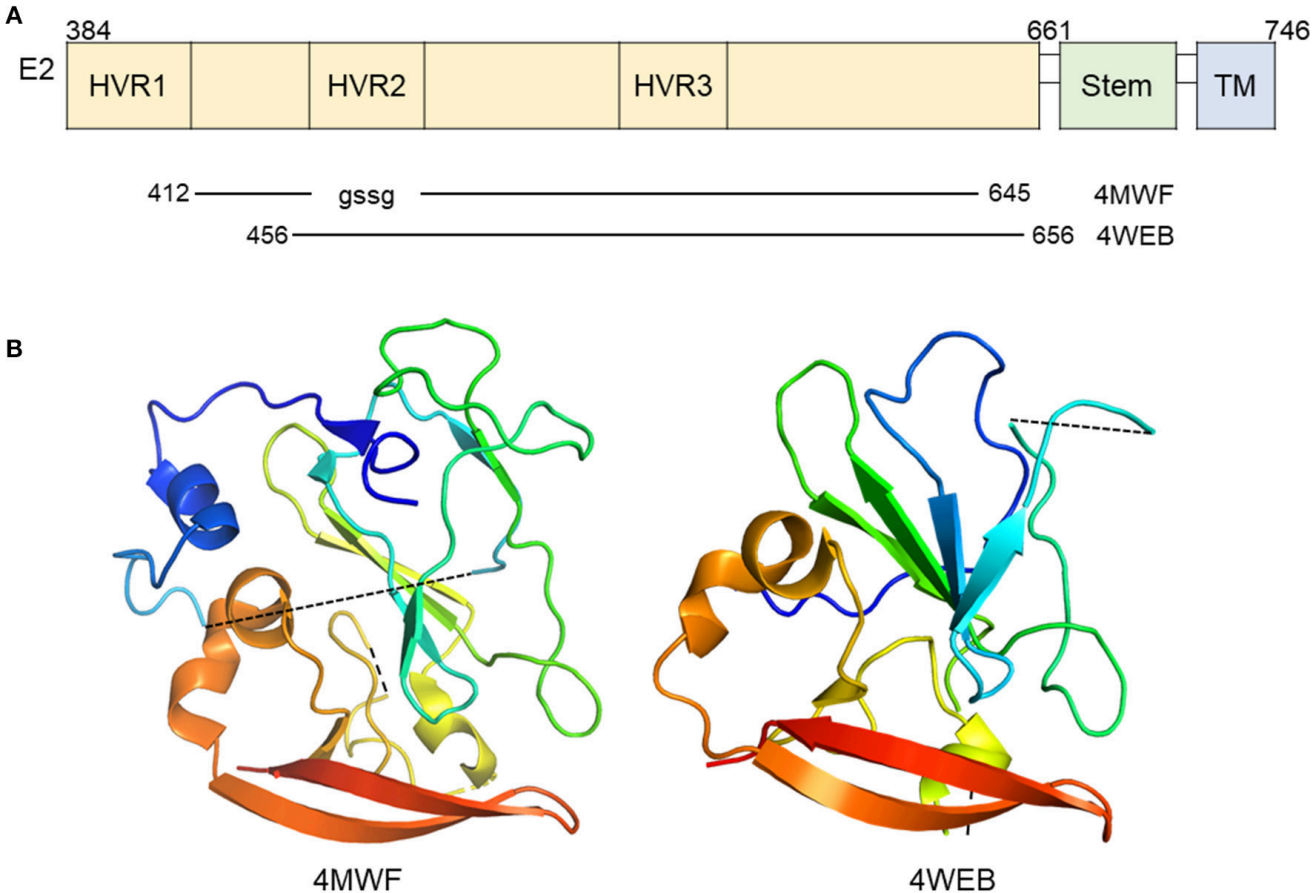
Crystal structure of E2 in complex with antibody. **(A)** Linear diagram of E2 glycoprotein. The crystallized E2 core constructs and PDB IDs are shown below the diagram. **(B)** Ribbon representation of E2 (PDB ID: 4MWF and 4WEB).

The functions of E2 have been more extensively studied relative to E1. E2 is responsible for mediating entry through interactions with several cellular receptors as mentioned above and is highly immunogenic (31–35). Two groups have published the structure of the core domain of E2 bound to Fabs (PDB ID: 4MWF and 4WEB) by X-ray crystallography (36, 37). The two studies employed a similar strategy with varying E2 expression constructs and antibodies for co-crystallization (Figure 2A). The 4MWF co-crystal was formed with E2 ectodomain (eE2) from HCV genotype 1a and a neutralizing, human Fab, AR3C that recognizing an N-terminal epitope in E2 and blocks E2-CD81 interaction. The eE2 in this structure does not contain hypervariable region 1 (HVR-1) and replaced HVR- 2 with a Gly-Ser-Ser-Gly linker. The 4WEB co-crystal was formed with eE2 from HCV genotype 2a, lacking the first 72 amino acids, and non-neutralizing Fab, 2A12, which binds a linear epitope at the C-terminus of eE2. Overall, both structures reveal a monomeric E2 with a globular nature (Figure 2B), unlike the class II fusion proteins that E2 was predicted to be similar to, and does not undergo major oligomeric or structural rearrangement upon exposure to low pH (37). Structural stability of the overall fold of the protein is provided by an extensive hydrophobic core and disulfide bonding. Follow-up alanine scanning studies mapped critically important E2 residues for neutralizing antibody recognition to core E2 stability elements and are in agreement with the published structures (38).

The ordered portions of E2 are primarily arranged in β-sheets stabilized by disulfide bonds and hydrophobic interactions; however, a majority of eE2 (62% of it in the case of 4MWF) is in flexible loops or completely unstructured (36). Hydrogen-deuterium exchange and limited proteolysis experiments implicate the first 72 amino acids of eE2 containing HVR1 and region between HVR1 and HVR2 as highly flexible (37). In the 4MWF structure, the AR3C antibody binds this strand and provides stabilization for crystal formation (36). X-ray diffraction data for HVR2 could not be obtained (37). Therefore, in the absence of a stabilizing antibody, that leaves approximately the first 100 amino acids of eE2, containing several glycosylation sites, flexible and solvent exposed. This region is involved in epitope shielding, SR-BI binding, CD81 binding, and neutralizing antibody recognition (31–33, 39–45).

## CD-81 binding site and neutralizing antibodies

**Figure 3:**
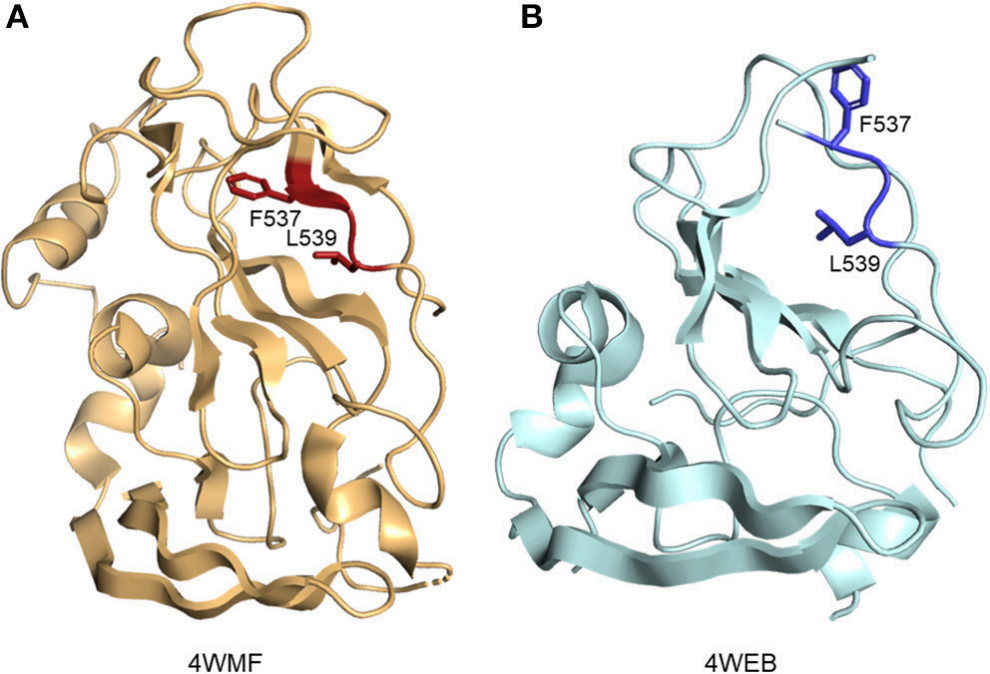
Ribbon representation of E2 hydrophobic residue positions in CD81 binding loop. **(A)** E2 residue F537 and L539 (side chains are shown as red sticks) in the presence of a stabilizing Fab fragment (not depicted) (PDB ID: 4MWF). **(B)** Solvent exposed positions of residue F537 and L539 (side chains are shown as blue sticks) (PDB ID: 4WEB).

Residues of E2 which form the CD81 binding site are found in clusters between aa412–446 and aa519–535 (termed the CD81 binding loop) of HCV genotype 1a strain H77 (36, 46, 47). Distant CD81 binding clusters are brought together by the overall fold of the protein. The two published eE2 structures, when compared, highlight the flexible nature of not only the CD81 binding loop, but the central immunoglobulin-like fold itself. In 4WEB, the CD81 binding loop is disordered, allowing hydrophobic residues to be solvent exposed. In the 4MWF structure, the CD81 binding loop is stabilized by a Fab fragment, bringing order to previously unstructured β-strand E and allowing residues such as F537 and L539 to be flipped into the hydrophobic core of the protein (Figure 3). In 2017, Vasiliauskaite et al. expanded on this observation by demonstrating that the hydrophobic residue positions and secondary structure in the CD81 binding loop of E2 were dependent on interactions with different neutralizing antibodies in both HCV pseudoparticles and cell culture-derived HCV particles (48). Given that the current evidence focus on binding to antibodies, the secondary structure of E2 bound to receptor CD81 may further reveal undescribed conformations.

**Figure 4:**
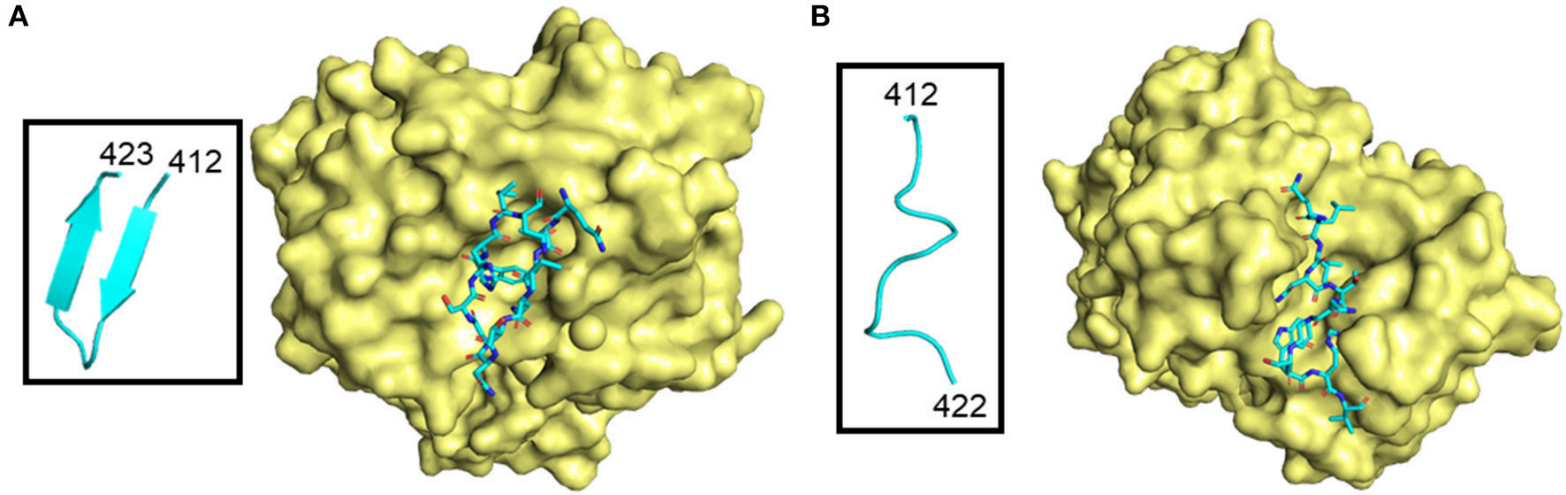
Different conformations of E2 412–423 peptide in complex with antibodies. The antibody surface is colored yellow, and the peptide is shown as sticks colored according to atom type (light blue, red, orange, and dark blue for carbon, oxygen, sulfur and nitrogen, respectively). E2 peptides are further highlighted in boxes and shown as light blue ribbon structures. **(A)** β-hairpin peptide in complex with HCV1 antibody (PDB ID: 4DGY). **(B)** “Open” conformation peptide in complex with neutralizing antibody 3/11 (PDB ID: 4WHY).

Many neutralizing antibody epitopes overlap CD81 binding residues of E2. For example, antibodies 3/11 and HCV1, as well as others, bind aa412–423, but recognize this flexible stretch of amino acids differently (32, 41, 49–52). The region adopts at least two different: an extended or “open” conformation or a β-hairpin (Figure 4) (50). Furthermore, the HCV1 antibody can bind from multiple angles, as visualized by electron microscopy, demonstrating not only local flexibility, but the long-reaching flexibility of the loop (53). Although the aa412–423 epitope is quite tempting for use in vaccine studies because of the cross-neutralizing antibody potential to functionally important residues, very few chronically infected HCV patients (<2.5%) produce a specific antibody response likely due to flexibility, and shielding by HVRs and glycans (45, 54–56). Recent studies seek to improve presentation of candidate epitopes and promote antigen recognition by the immune system using an engineered, cyclic immunogen. Initial data shows a designed E2 cyclic immunogen produced a strong antibody response in mice, whose serum was then used to successfully neutralize HCV infection in culture experiments (57). Further research will determine whether engineered derivations of this epitope will be useful in the pursuit of a viable HCV vaccine.

## HCV virion-associated E1 and E2

Structural information of the HCV virion is lacking, in sharp contrast to the closely related flaviviruses and alphaviruses. Obtaining a high-resolution, three dimensional structure of the HCV virion is difficult due to the low level of virus production in cell culture systems and the inherent heterogeneity of the particles owing to its association with apolipoproteins. Cryo-electron microscopy and tomography of HCV virions show spherical particles of highly heterogeneous size (40–100 nm in diameter). The particles displayed no obvious symmetry, no evidence of continuous membrane bilayer, and are covered by electron-dense material; although, the inherent low resolution of the electron micrographs may mask certain features (16). These findings perhaps call into question the hypothesis that HCV adopts a classical, icosahedral scaffold in which its two envelope glycoproteins anchor to the host cell-derived, double-layer lipid envelope. The lack of symmetry and membrane bilayer highlights the unique nature of the HCV virion relative to the other flaviviruses.

Higher-order aggregates of E1 and E2 on secreted virus particles appear to be covalently bonded, whereas non-covalently associated E1/E2 has been detected in the ER (58–60). At the moment, composition and structural information are unavailable for these higher order aggregates; however, one may glean insight from available structural information on E1 and E2 (28, 36, 37). The asymmetric unit of the nE1 structure contains six monomers stabilized by a series of intramolecular and intermolecular disulfide bonds (Figure 1C). It is possible that some or all of the intermolecular disulfides in the nE1 structure may be relevant to the higher order structures seen on the virion. The two eE2 structures are highly similar, with an RMSD of less than 0.8 Å for similar carbon-alpha positions with most of the differences located in loop regions. Interestingly, there are discrepancies in the disulfide bonding pattern in these regions. The overall fold of E2 core is unlikely to change in the virion, owing to its extensive hydrophobic core and three disulfide bonds formed between secondary structure elements.

The current structures available for E1 and E2 may reflect the immature forms of the proteins after initial synthesis and during virion assembly. Our hypothesis is that the folds found in these structures would be present on the virion, with the higher order aggregates formed via disulfide bonding through cysteines found in loop regions or within the core domains. During virion assembly and maturation, these core domains fold and higher order structures begin to form within the heterodimer or through interactions with other factors. The environment of the ER and Golgi apparatus during egress is oxidizing and compatible with disulfide bond formation and reshuffling, permitting the formation of the disulfide-linked aggregates. This maturation may contribute to the acid-resistance of extracellular HCV virions and have implications for the mechanisms of entry. Indeed, cell surface-bound HCV needs to be incubated for prolonged periods at 37°C for low-pH-mediated entry to occur (61). This suggests that post-binding events are required to prime the HCV envelope proteins for fusion.

## Conclusion

Targeting a conserved epitope with known functional relevance is absolutely essential for production of a broadly neutralizing antibody. Structure-based vaccine design and innovative thinking with regard to stabilization of epitopes will be necessary to forward HCV vaccine efforts. Many of the vaccine studies in the past decade have been done with recombinant HCV E2 or E1E2; however, a majority of the human antibody responses were against E2 HVR1 and ultimately unsuccessful due to the high mutation rate in the region (62). The highly disordered and flexible nature of HCV E2 is a complicating factor to intelligent vaccine design. Not only is local flexibility seen between the same epitope partnered with different antibodies, but large portions of E2 are disordered and variably-sequenced between genotypes (i.e., the HVRs). The described structures of E1 and E2 are only representative of their respective serotypes and may or may not be representative of the many variable HCV isolates that exist. Within the HCV patient population, many circulating isolates are highly resistant to known broadly neutralizing antibodies, and many mutations that allow for resistance to neutralizing antibody recognition have been described (63, 64). Furthermore, inherently flexible, long-chain glycans are responsible for shielding targeted neutralizing antibody binding sites.

Available DAA treatments for HCV are undoubtedly necessary for infected patients; however, taking into account the rate at which high-risk groups are being infected with HCV, a vaccine is an imperative for preventative treatment. In order to achieve this goal, researchers must overcome the problem of HCV which uses an almost shapeshifting mechanism to evade immune detection: shrouding itself with a coat of apolipoproteins, flexibility, and hyper variability. HCV E2 has evolved to maintain a balance between the order of disulfide bonds and hydrophobic interactions necessary to form the overall protein fold, and the flexible chaos which allows the virus to replicate while evading the host immune response.

## Author contributions

SY wrote the paper. YW and JM generated the figures. SY, YW, and JM participated in the critical review and revision of the paper.

### Conflict of interest statement

The authors declare that the research was conducted in the absence of any commercial or financial relationships that could be construed as a potential conflict of interest.
